# Improving the Yield of Histological Sampling in Patients With Suspected Colorectal Cancer During Colonoscopy by Introducing a Colonoscopy Quality Assurance Program

**DOI:** 10.4021/gr334w

**Published:** 2011-07-20

**Authors:** Ahmed Gado, Basel Ebeid, Aida Abdelmohsen, Anthony Axon

**Affiliations:** a Department of Medicine, Bolak Eldakror Hospital, Giza, Egypt; b Department of Tropical Medicine and Infectious Diseases, Banysweef University, Banysweef, Egypt; c Depertment of Public Health, National Research Center, Giza, Egypt; d Department of Gastroenterology, The General Infirmary at Leeds, Leeds, United Kingdom

**Keywords:** Colorectal cancer, Biopsy, Yield of histological sampling

## Abstract

**Background:**

Masses discovered by clinical examination, imaging or endoscopic studies that are suspicious for malignancy typically require biopsy confirmation before treatment is initiated. Biopsy specimens may fail to yield a definitive diagnosis if the lesion is extensively ulcerated or otherwise necrotic and viable tumor tissue is not obtained on sampling. The diagnostic yield is improved when multiple biopsy samples (BSs) are taken. A colonoscopy quality-assurance program (CQAP) was instituted in 2003 in our institution. The aim of this study was to determine the effect of instituting a CQAP on the yield of histological sampling in patients with suspected colorectal cancer (CRC) during colonoscopy.

**Method:**

Initial assessment of colonoscopy practice was performed in 2003. A total of five patients with suspected CRC during colonoscopy were documented in 2003. BSs confirmed CRC in three (60%) patients and were nondiagnostic in two (40%). A quality-improvement process was instituted which required a minimum six BSs with adequate size of the samples from any suspected CRC during colonoscopy. A total of 37 patients for the period 2004-2010 were prospectively assessed.

**Results:**

The diagnosis of CRC was confirmed with histological examination of BSs obtained during colonoscopy in 63% of patients in 2004, 60% in 2005, 50% in 2006, 67% in 2007, 100% in 2008, 67% in 2009 and 100% in 2010. The yield of histological sampling increased significantly (*p*<0.02) from 61% in 2004-2007 to 92% in 2008-2010.

**Conclusion:**

The implementation of a quality assurance and improvement program increased the yield of histological sampling in patients with suspected CRC during colonoscopy.

## Introduction

Histopathology plays a critical role in gastrointestinal practice [1]. Masses discovered by clinical examination, imaging or endoscopic studies that are suspicious for malignancy typically require biopsy confirmation before treatment is initiated. The role of biopsy is to exclude the presence of benign lesions that may mimic malignancy clinically and if malignant tumor is present, to determine the histologic type. Even when direct access to the tumor is possible, biopsy specimens may fail to yield a definitive diagnosis if the lesion is extensively ulcerated or otherwise necrotic and viable tumor tissue is not obtained on sampling. The diagnostic yield is improved when multiple biopsy samples (BSs) are taken [2].

Colorectal cancer (CRC) is the forth most commonly diagnosed cancer and the second leading cause of cancer related death in the United States [3]. Once CRC has developed, colonoscopy also has an important role in the diagnosis and subsequent disease management. During colonoscopy, every effort should be made to obtain a tissue diagnosis when encountering polyps, mass lesions, or colonic strictures. There are very few well-designed, prospective studies that address the optimal number of endoscopic biopsy specimens necessary to diagnose CRC. In a prospective study of 60 patients with malignant colonic lesions confirmed by surgical pathology, four biopsy specimens obtained during colonoscopy yielded a diagnosis of CRC in 68%, whereas six biopsy specimens yielded a diagnosis in 78%. There was no additional diagnostic yield from obtaining more than six biopsy specimens. In cases where endoscopic biopsy specimens are nondiagnostic and cancer is highly suspected, clinicians should consider obtaining a second opinion from an expert pathologist and/or performing repeat colonoscopy for additional tissue sampling. Surgery is indicated for suspicious lesions with nondiagnostic biopsy specimens [3].

Bolak Eldakror Hospital is a secondary-care governmental hospital in Giza, Egypt. The gastrointestinal endoscopy unit was founded in 1999. A colonoscopy quality-assurance program (CQAP) was instituted in 2003 [4-8]. Accordingly, the quality indicators developed by the American Society of Gastrointestinal Endoscopy and the British Society of Gastroenterology were implemented [9, 10]. For easy application, quality indicators were identified for five major groups: patients, procedures, endoscopists, assistant staff and equipment. Process or outcome indicators were used in evaluating and monitoring the quality of endoscopic procedures. The present study was undertaken to determine the effect of instituting a CQAP on the yield of histological sampling in patients with suspected CRC during colonoscopy.

## Material and Method

All patients with suspected CRC during colonoscopy were included in the study. Patients underwent colonoscopy by a gastroenterologist using a videocolonoscope (Olympus CF-230L/I). Standard size biopsy forceps (Olympus FB-24U-1) were used to take pinch biopsies. BSs were collected on filter papers before placed in a vial with 10% formalin. BSs were examined by two separate pathologists. Tumor markers were not available. CT scan was performed in all patients followed by surgery and histological examination of resection specimens. All patients were referred to the National Cancer Institute for further management.

Initial assessment of colonoscopy practice was performed in 2003. A total of five patients with suspected CRC during colonoscopy were documented in 2003. BSs confirmed CRC in three (60%) patients and were nondiagnostic in two (40%). A quality-improvement process was instituted which required a minimum six BSs from any suspected CRC during colonoscopy. Endoscopist should look to BSs on filter paper to ensure adequate size of samples. If any BS is tiny or contains blood, mucus or necrotic tissues it should be replaced by another adequate size sample of tumor tissue. Findings were compared with the resection specimen results. Between 2004 and 2010, annual quality-assurance reports were transmitted to an independent experienced endoscopist with a particular interest in quality assurance for comment and advice.

A total of 37 patients referred from outpatient clinic, medical department and other hospitals for the period 2004-2010 were prospectively assessed. CRC was suspected during colonoscopy in all the patients. The yields of histological sampling during colonoscopy were assessed over a period of sevenyears.

Statistical analysis: Data entry, tabulation and analysis were done using Statistical Package for the Social Sciences (SPSS) program for Window version 13. Descriptive data are presented as percentage and chi-square test was performed to detect the differences between two categorical groups.

## Results

A total of 37 patients with suspected CRC during colonoscopy were documented during 2004-2010. One patient was excluded because BSs result was not available. A total of 36 patients were included in the study. Out of these, 50% were women and 50% were men. Mean age was 47years (range: 16-80years). The indications for the procedures were rectal bleeding in 28%, chronic diarrhea in 22%, intestinal obstruction in 19%, abdominal mass in 14%, anemia in 8%, CRC follow up in 3%, constipation in 3% and a lesion identified on another diagnostic procedure which required further evaluation in 3%. Colonoscopy revealed colorectal mass in all the patients. Thirty six percent of lesions were located in the sigmoid colon, 19% in the rectum, 14% in the descending colon, 11% in the ascending colon, 8% in the cecum, 6% in hepatic flexure and 6% in the transverse colon. The diagnosis of CRC was confirmed with histological examination of BSs obtained during colonoscopy in 26 (72%) patients and in 10 (28%) patients BSs were nondiagnostic (negative). Adenocarcinoma was diagnosed in 61% of patients, mucoid carcinoma in 6%, signet ring cell carcinoma in 3% and serrated adenoma with intramucosal carcinoma in 3%. Colonoscopy was repeated in three (30%) of the patients with negative biopsies to take more biopsies while the other seven (70%) patients underwent surgery without repeating colonoscopy. The additional BSs did not identify any cancer. All patients underwent a colorectal resection. Patients with negative biopsies underwent colorectal resection based of clinical assessment, colonoscopic appearance and suggestive CT scan findings. CRC (adenocarcinoma) was diagnosed in all resection specimens of patients with negative biopsies.

**Figure 1:**
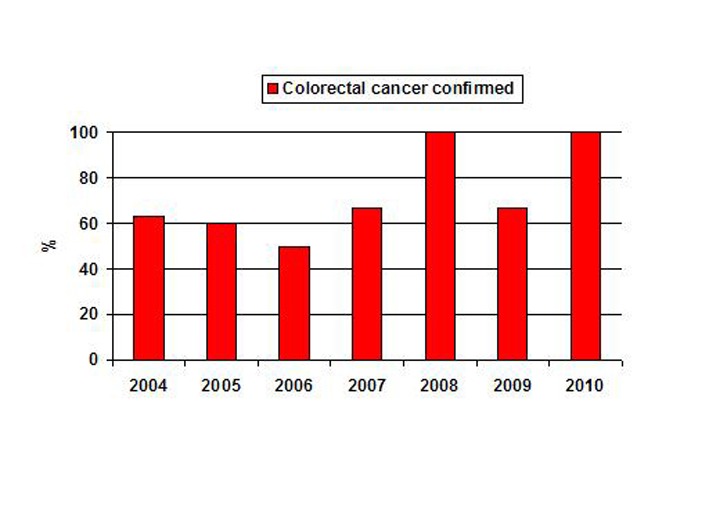
The yield of histological sampling in patients with suspected colorectal cancer during colonoscopy among studied years.

A total of 36 patients with suspected CRC during colonoscopy were assessed: eight patients in 2004, five in 2005, four in 2006, six in 2007, four in 2008, three in 2009 and six in 2010. The diagnosis of CRC was confirmed with histological examination of BSs obtained during colonoscopy in five (63%) patients in 2004, three (60%) in 2005, two (50%) in 2006, four (67%) in 2007, four (100%) in 2008, two (67%) in 2009 and six (100%) in 2010 (Fig. 1). The yield of histological sampling increased significantly (*p*<0.02) from 61% in 2004-2007 to 92% in 2008-2010 (Table 1).

**Table 1:** The Yield of Histological Sampling in Patients With Suspected Colorectal Cancer During Colonoscopy Among Studied Years

Studied year	Colorectal cancer confirmed	Negative biopsy specimens	Total
	*n* (%)	*n* (%)	*n* (%)
2004-2007	14 (60.87)	9 (39.13)	23 (100)
2008-2010	12 (92.31)	1 (7.69)	13 (100)

χ^2^ = 4.09, P< 0.02: significant.

## Discussion

The effectiveness of colonoscopy depends on the technical quality of the procedure [11]. The goal of maintaining and enhancing the quality of services should be addressed by a continuous process of measuring aspects of endoscopic performance [9]. Continuous quality improvement has been recommended by professional societies as a part of every colonoscopy programme [12]. A CQAP was instituted in 2003 in our institution. Process or outcome indicators were used to assess the quality of endoscopic procedures and monitor performance. Assessment was performed by scoping and evaluating our endoscopy service. Benchmarking was used to detect shortcomings and deviations from standards. A continuous quality improvement process was implemented, this involved changing some of our management practices and the way we performed our endoscopic procedures. Cecal intubation rate, quality of bowel preparation, patient satisfaction and polypectomy practice were improved [6, 7]. Infection control of endoscopies was also improved [5].

An important issue to clinical practice is the accuracy of the gold standard (pathology) that affects the clinical outcome [13]. The diagnostic yield of histopathology depends on several factors including the pathologist’s level of experience, and also the quality of BSs and sampling errors [1]. The quality of samples is influenced by a variety of elements such as the size and shape of biopsy forceps, the endoscopist’s level of experience and the number of samples [1]. We previously reported improving the detection rate of microscopic colitis after modification of performance of biopsy in patients with chronic diarrhea [8]. Colonoscopy has an important role in the diagnosis of CRC. During colonoscopy, every effort should be made to obtain a tissue diagnosis when encountering polyps, mass lesions, or colonic strictures. Accurate diagnoses can be difficult from a small biopsy. Repeating endoscopy to obtain more biopsies will increase the cost and can affect patient satisfaction. In this study performance of biopsy in patients with CRC was assessed in 2003 in our institution. BSs up until then were taken randomly (without a definitive number) and confirmed CRC in only 60% of patients with suspected CRC during colonoscopy in 2003. The quality improvement process instituted required a minimum six BSs from any suspected CRC during colonoscopy and BSs should be adequate in size. The new protocol was adhered to. Consequently the diagnosis of CRC confirmed with histological examination of BSs obtained during colonoscopy increased significantly (*p*<0.02) from 61% in 2004-2007 to 92% in 2008-2010. In our study 8% of CRC was missed by biopsy in 2008-2010. It is reported that although four to six biopsies is recommended yet even this will miss 8-10% of colorectal cancers [15]. Colonoscopy was repeated in 30% of patients with negative biopsies to take more biopsies. The addition of more BSs did not increase the diagnostic yield. This is similar to the previous reports that there were no additional cancers identified by taking more biopsies (eight or ten total) [3, 14]. Several different techniques were documented to increase the yield of histological sampling e.g. brush cytology, jumbo forceps and disposable forceps. Colonoscopic brushing cytodiagnosis is a sensitive technique for the detection of CRC [15]. The use of brush cytology improves the yield of tissue diagnosis considerably when added to the biopsy technique [15]. Brush cytology should be used with biopsy to get maximal yield (97% accuracy), especially in areas of strictures or obstruction [16]. Biopsies can also be taken using “jumbo” forceps. These jumbo forceps do not fit though the biopsy channel of a standard diagnostic endoscope and require a special therapeutic instrument not available in all settings. The specimens obtained with the jumbo forceps are larger in size than the standard endoscopy forceps but are also associated with slightly higher risk of bleeding [17]. A greater yield was also documented from disposable forceps. This is most likely attributable to the fact that the disposable forceps are, on average, sharper than the reusable forceps. Reusable forceps can become dull with repeated use and mechanical cleaning. In addition, the hinge and cable mechanism of the disposable forceps are likely to operate more smoothly and reliably and are less likely to malfunction because they are used only once. A disadvantage of the disposable forceps is cost [17].

The implementation of a quality assurance and improvement program increased the yield of histological sampling in patients with suspected colorectal cancer during colonoscopy. This study is performed in a setting of self-evaluation and evaluates quality using an approach based on measurement and comparison. It allowed us to detect certain shortcomings and deviations from standards and to implement a quality improvement process. The quality assurance program is a part of an overall program designed to improve quality of endoscopy practice in our unit [4-8]. The major drawbacks of the present study are it involved a single centre and had a low volume of patients.
